# Distal transradial access: a review of the feasibility and safety in cardiovascular angiography and intervention

**DOI:** 10.1186/s12872-020-01625-8

**Published:** 2020-08-05

**Authors:** Gaojun Cai, Haomin Huang, Feng Li, Ganwei Shi, Xiaolong Yu, Lei Yu

**Affiliations:** 1 Department of Cardiology, Wujin Hospital Affiliated with Jiangsu University, the Wujin Clinical College of Xuzhou Medical University, Changzhou City, Jiangsu Province China; 2 Science and Education Section, Wujin Hospital Affiliated with Jiangsu University, the Wujin Clinical College of Xuzhou Medical University, Changzhou City, Jiangsu Province China

## Abstract

**Background:**

Transradial access (TRA) has been considered as the default choice in cardiac catheterization. Although infrequent, vascular complications of this approach remain. Recently, the distal transradial approach (dTRA) in cardiac catheterization was reported by interventionalists.

**Methods:**

We retrieved the relevant literatures and reviewed the safety and feasibility of this novel approach in cardiac catheterization.

**Results:**

The dTRA for cardiac intervention has superior safety and satisfaction. As a novel approach for cardiac catheterization, access related complications should also be considered by operators, such as RAO, radial spasm, bleeding and haematoma, and injury of the superficial branch of the radial nerve.

**Conclusions:**

The dTRA in cardiovascular angiography and intervention was safe and feasible.

## Background

Transradial access (TRA) has been considered as the default choice in cardiac catheterization because of the decreased access site complications, increased patients comfort, early mobilization, etc. [1]. Compared with femoral access, radial access has a similar procedural success rate and is associated with a significantly lower risk for all-cause mortality and major adverse cardiovascular events [2]. This benefit also exists in patients presenting with acute coronary syndrome [3]. In 2018, it was recommended by the ESC/EACTS guidelines as the preferred approach in myocardial revascularization procedures [4]. Although infrequent, vascular complications associated with this approach remain, such as radial artery injury, radial artery spasm, radial artery occlusion (RAO), pseudoaneurysm, arteriovenous fistula, nerve damage and complex regional pain syndrome [5]. Although the transulnar artery approach, as an alternative approach, has the same safety and efficacy in cardiac catheterization as TRA, this access is not preferred because of some drawbacks, such as requiring more wrist rotation during the procedure, which increases the patient discomfort [6].

In recent years, the distal transradial approach (dTRA), as an alternative approach for cardiac catheterization, has gradually become familiar to cardiologic interventionalists. In the past 2 years, an increasing number of studies have focused on the feasibility and safety of the dTRA approach for coronary angiography (CAG) and percutaneous coronary intervention (PCI) in different countries [7–15]. However, as a new approach, it also has some disadvantages. The present review reported the safety and feasibility of dTRA in cardiovascular angiography and intervention.

## History of dTRA

TRA for CAG and PCI was first reported in 1989 by Campaeu and in 1993 by Kiemeneij, respectively [16, 17]. Currently, TRA has become the most favourable and the default approach for cardiac catheterization. After three decades of application, several kinds of complications have been reported [5]. Among them, RAO is one of the most important complications. However, the incidence of RAO may be seriously underestimated due to the absence of symptoms. As a matter of fact, the incidence of RAO is not rare, ranging greatly from < 1 to 33% according to the results in recent studies [18]. Although most cases of occlusion are occult and lack of ischemic syndrome, RAO prohibits future utilization of the radial artery, including repeat access for staged or repeat PCI, establishing arteriovenous fistula in chronic renal dysfunction, its use as the grafting vessel in coronary artery bypass graft, etc. With increases in the average life expectancy and with improvements in medical conditions, the possibility of multiple interventions via the radial artery is increasing. In addition, when encountering right radial artery tortuosity, vasospasm or occlusion, left internal mammary graft angiography, the left radial artery is forced to be chosen as the access vessel. The hand of the patient is restricted in a supine or semiprone position during the operation, which increases the discomfort of patient. More serious situations include the cases of an obese patient and a short operator, which may greatly increase discomfort not only in the patient but also in the operator. Therefore new puncture approaches are expected with decreased vessel complications, increased patient comfort and preservation of the traditional radial artery as much as possible.

dTRA was introduced by anaesthesiologists for perioperative patient monitoring several decades ago [19]. In 2011, Babunashvili A et al. [20] first described their experience with retrograde recanalization of the occluded radial artery via dTRA. In 2017, Kiemeneij [21] shared the experience of 70 selected patients out of 118 patients who underwent cardiac catheterization via the left distal radial artery (DRA) at the anatomic snuffbox (AS) in EuroIntervention. The methods of puncture, results of procedural data and complications were described in detail. Since then, an increasing number of studies have reported the feasibility and safety of dTRA for CAG and PCI. In the ClinicalTrials databases (www.clinicaltrials.gov), a total of 15 studies were registered to explore the clinical value of dTRA in cardiac catheterization (last search was updated to February 10th, 2020). The search terms used were: “distal radial artery, distal transradial artery, anatomical snuffbox, snuffbox, radial fossa, fovea radialis, fossa radialis”. The detailed information was listed in Table 1. After comprehensively searching on PubMed, Embase, Google Scholar databases and combining with manual searches of references, a total of 25 papers with sample sizes greater than 20 reported the application of dTRA in cardiac catheterization, including case-series as well as non-randomized and randomized studies, after removing duplicate data (Table 2 )[21–45].

**Table 1:** Characteristics of clinical trails registered in ClinicalTrails databases

Identifier	Title	Country	Study type	Estimated Enrollment	Time	Status
NCT03292367	The Procedural Success and Complication Rate of the Left Distal Radial Approach	Korea	Cohort	200	October 2017–February 2018	Completed
NCT03373565	Snuff-box Deep Palmar Arch Artery Versus Radial Angiography	Iran	Randomized	200	July 2017–December 2017	Unknown
NCT03486470	Comparison of Two Radial Artery Segments Related to “Old-Fashioned” Radial and New Snuff Box Vessel Approach by US	Bulgaria	Cross-Sectional	520	April 2018–August 2018	Not yet recruiting
NCT03611725	Comparison of Success Rate Between Distal RadialApproach and Radial Approach in STEMI	Korea	Randomized	352	August 2018–October 2020	Recruiting
NCT03789279	Observational Study of Hand Function After DistalTransradial Access for Angiography	United Kingdom	Cohort	40	Janaury 2019- June 2020	Active, not recruiting
NCT03794687	The Distal (SnUffbox) Radial artERy Access for Coronary Angiography and Interventions (SUPER-Prospective)	United States	Cohort	100	April 2019-Janaury 2020	Recruiting
NCT03863652	Optimal Hemostasis Duration for Percutaneous Coronary Intervention Via Snuffbox Approach (HEMOBOX)	Korea	Cohort	200	December 2019–February 2020	Recruiting
NCT03948165	Distal Transradial Access for Coronary Angiography and Percutaneous Coronary Intervention.	Mexico	Cohort	100	November 2017–December 2018	Completed
NCT03986151	Anatomical sNuffbox for Coronary anGiography and IntervEntions (ANGIE)	Greece	Randomized	774	June 2019–February 2021	Recruiting
NCT04023838	Randomized Comparison of Radiation Exposure in Coronary Angiography Between Right Conventional and Left Distal Radial Artery Approach	Korea	Randomized	100	August 2019–December 2020	Not yet recruiting
NCT04080700	Korean Prospective Registry for Evaluating the Safety and Efficacy of Distal Radial Approach (KODRA)	Korea	Cohort	5000	September 2019–December 2020	Recruiting
NCT04125992	Distal Radial Artery vs. Forearm Radial Artery For Cardiac Catheterization	Palestine	Randomized	200	December 2018-Janaury 2020	Recruiting
NCT04171570	DIStal Versus COnventional RADIAL Access for Coronary Angiography and Intervention	Multi-countries	Randomized	130	November 2019–November 2020	Recruiting
NCT04194606	CORonaRy Angiography and intErventions Via Distal vs Proximal aCcess	Germany	Randomized	500	December 2019–December 2021	Not yet recruiting
NCT04238026	Distal Radial Artery Approach to Prevent Radial Artery Occlusion (DAPRAO)	Mexico	Randomized	268	May 2019–March 2020	Recruiting

**Table 2:** Characteristics of researches on the feasibility of DRA in cardiac catheterization

Year	First author	Country	Study design	Access site	Number (n)	Age (Mean, y)	Male (%)	Success rate (%)	ACS (%)	PCI (%)	≥6F Sheath (%)	Right (%)	Diameter (Mean, mm)
2017	Kiemeneij F [21]	Netherlands	Case-series	DRA	70	68	79	89	NA	36	58	0	NA
2018	Soydan E [22]	Turkey	Case-series	DRA	54	59.3	80	100	31.4	8.5	100	0	NA
2018	Valsecchi O [23]	Italy	Case-series	DRA	52	68	82	90	9.6	48.1	98.1	84.6	2.22
2018	Kim Y [24]	South Korea	Retrospective	DRA	150	65.9	71.2	93.3^c^	43.9	27.3	100	0	2.57
2018	Lee JW [25]	South Korea	Prospective	DRA	200	66.1	66	95.5	31	43.5	33.0	0	2.41^a^/2.36^b^
2018	Ziakas A [26]	Greece	Case-series	DRA	49	64	63.3	91.8^c^	24.5	18.2	79.6	100	NA
2018	Babunashvili A [27]	Russia	Retrospective	DRA	1320	63.2	68.1	99.7	NA	47.6	45.6	80.9	2.13
2018	Al-Azizi KM [28]	America	Case-series	DRA	61	70	75.4	98.4	73.7	55.7	86.9	0	NA
2018	Flores EA [29]	Georgia	Retrospective	DRA	200	64	69	95.5	51.0	28	NA	85	NA
2019	Mizuguchi Y [30]	Japan	Retrospective	DRA	228	71.1	71.1	100	NA	33.8	29	88.6	2.4
2019	Norimatsu K [31]	Japan	Retrospective	DRA	74	70	78	92	8	40	23	84	2.6
2019	Wretowski D [32]	Poland	Case-series	DRA	218	68	65.14	89.4	19.72	36.2	56.9	51	NA
2019	Gasparini GL [33]	Italy	Prospective	DRA	47	67.8	75.6	82.9	NA	100	100	NA	NA
2019	Uddin MJ [34]	Bangladesh	Case-series	DRA	200	52.3	75	98.5	NA	36	NA	100	NA
2019	Amin MR [35]	Bangladesh	Case-series	DRA	100	NA	NA	98	NA	NA	NA	NA	NA
2020	Yu WW [36]	China	Case-series	DRA	92	69	62.0	95.7	48.9	42.4	100	95.7	1.71
2017	Kaledin A [37]	Russia	Retrospective	DRA^d^	2 696	NA	NA	97.7	NA	NA	99.6	NA	2.4
				TRA	2781	NA	NA	96	NA	NA	99.3	NA	2.7
2018	Coughlan JJ [38]	Iran	Non randomized control study	DRA	47	61	83	100	NA	0	10.6	0	NA
				TRA	47	61.8	74.5	100	NA	0	17.1	0	NA
2018	Roghani-Dehkordi F [39]	Iran	Cross-sectional study	DRA	235	NA	76.5	94.1	NA	28.9	NA	NA	NA
				Trans-palmar	175	NA	76	90.8	NA	18.2	NA	NA	NA
2018	Koutouzis M [40]	Greece	Randomized control study	DRA	100	63.8	74	70	NA	NA	NA	NA	NA
				TRA	100	62.8	77	98	NA	NA	NA	NA	NA
2018	Gajurel RM [41]	Nepal	Prospective study	DRA	82	57.7	58.5	97.6	NA	24.3	65.8	100	NA
				TRA	82	57.2	53.6	96.4	NA	39.0	71.9	100	NA
2019	Aoi S [42]	America	Retrospective	DRA	202	69.2	64.9	99.5	NA	44.6	NA	88.1	NA
				TRA	206	68.8	62.6	99	NA	44.7	NA	90.8	NA
2019	El Tahaan M [43]	Egypt	Non randomized control study	DRA	50	53.42	70.0	92	NA	36	NA	44	NA
				TRA	50	54.07	72.0	100	NA	34	NA	44	NA
2019	Mori S [44]	Japan	Prospective	DRA	43	NA	NA	92.9	NA	35.9	NA	NA	NA
				TRA	53	NA	NA	94.3	NA	41.1	NA	NA	NA
2019	Vefalı V [45]	Turkey	Non randomized control study	DRA	102	60.89	70.6	95.1	NA	23.53	NA	15	2.05
				TRA	103	59.84	68	96.1	NA	24.27	NA	18	2.32

*Abbreviations*: *NA*, non available, *ACS* acute coronary syndrome, *PCI* percutanous coronary intervention

^a^left side

^b^right side

^c^puncture success rate

^d^patients undergoing coronary intervention, including both anatomic snuffbox and dorsum of hand

## Anatomy of the DRA

Normally, the radial artery, originating in the cubital fossa, descends along the lateral side of the forearm to the wrist. After giving rise to the superficial palmar branch, which forms the superficial palmar arch with the ulnar branch, it course around the styloid process of radius to the dorsal side of the hand, and then runs forward on the bottom of the AS. Finally, it crosses the surface of the metacarpal bones and connects with the distal part of the ulnar artery, completing the deeper palmar arch, which is formed mainly by the terminal part of the radial artery. There are rich collateral anastomotic branches between the two palmar arches. Traditionally, the radial artery puncture site has been considered to be from the distal third of the forearm to the rasceta because of the superficial position that facilitates puncture and haemostasis.

**Fig. 1:**
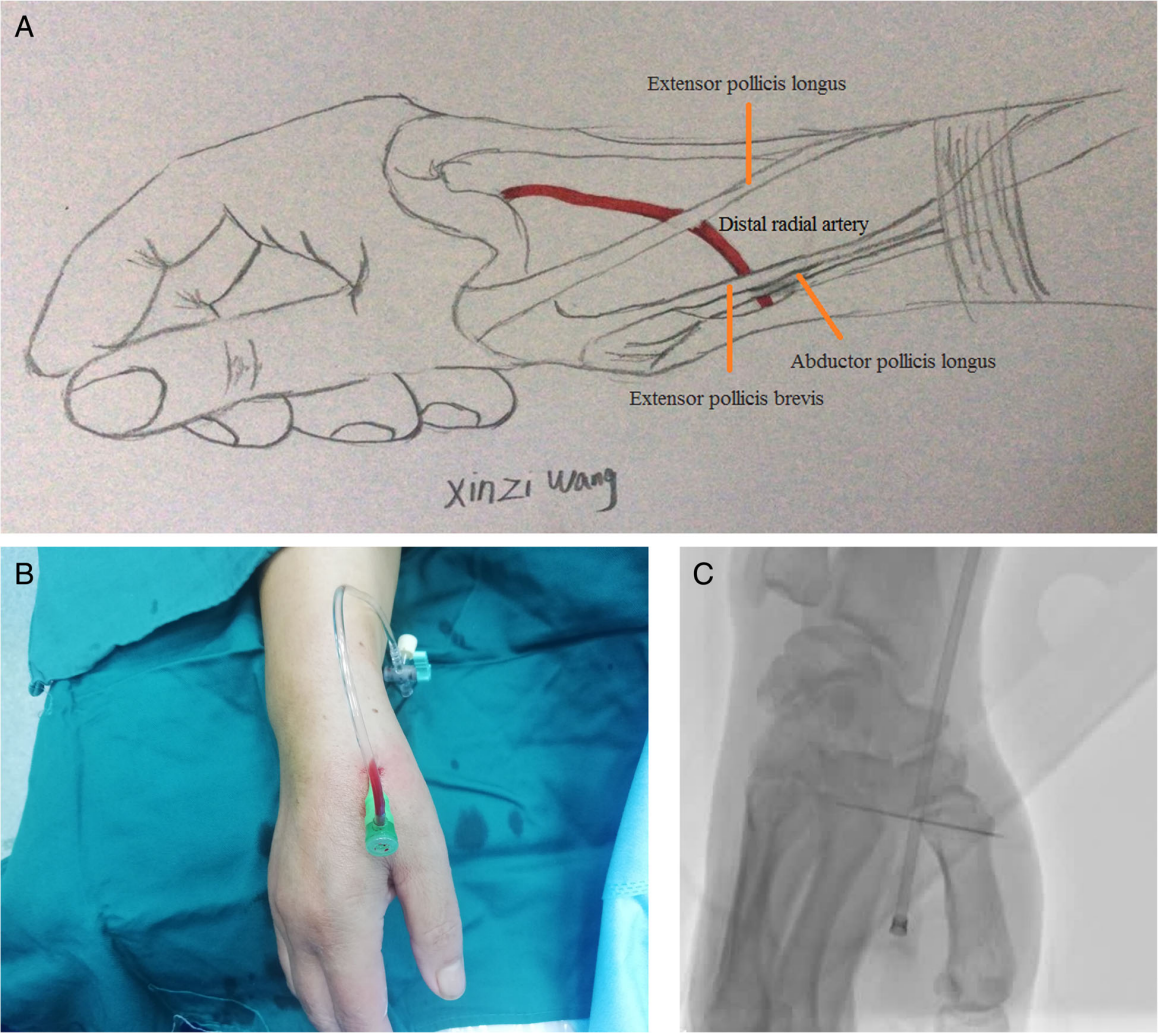
Anatomy of DRA (**a**. Sketch of DRA; **b**. 6Fr Sheath implanted into DRA; **c**. Location of the puncture site under X-ray)

The AS, a triangular-shaped space, is located on the lateral side of the dorsum of the wrist and is clear when the thumb is stretched out. The AS is bound by the tendons of the extensor pollicis brevis and the abductor pollicis longus on the lateral side and the tendon of the extensor pollicis longus on the medial side. The base of the triangle is formed by the styloid process of the radius. The trapezium and scaphoid bones consist of the bottom of the AS. In the region of the AS, the radial artery is prone to palpitation and becomes the superior site for puncture because of its superficial position and bony basement. The dTRA includes the AS site and very distal radial artery, which is in the vertex of the angle between the tendon of the extensor pollicis longus and the second metacarpal bone (Fig. 1) [30, 37]. However, most interventionalists are prone to choose the AS region as the dTRA site. Cardiac interventionalists who being used to femoral artery access, are prefer to select the left DRA due to the anatomy of the radial-aortic passage resembling that of the femoral-aortic passage [46]. However, operators who are accustomed to the right radial artery approach tend to adopt right DRA access.

## Feasibility

It is undeniable that, as a novel technique, it faces some challenges. The first and most important challenge is the success rate of puncture. The diameter of the artery in the AS is smaller than that in the forearm. Additionally, the artery is more tortuous, which may decrease the success rate of puncture. Therefore, many operators believe that there is a certain learning curve and have suggested that this procedure should be performed by well trained interventionalists. As reported in the 24 researches with sample sizes greater than 20, the success rate varied greatly and ranged from 70 to 100% (Table 2). In the two largest studies, the success rate was 99.7% in 1631 patients and 97.7% in 2696 patients [27, 37]. Interestingly, only one randomized study reported that the success rate in the AS was less than 80%, which was significantly lower than that in the TRA [40]. However, we should realize that some factors may affect the results. The first is the criteria of success. In some studies, success was defined when the needle was successfully punctured into the vessel, whereas in others, success was defined as sheath insertion. For example, Kim Y reported that the success rate was 93.3% according to the previous definition but was only 88.0% according to the latter definition [24]. Ziakas A et al. [26] also obtained similar results. The methods of puncture also affect the success rate. Some operators made use of the “puncturing the anterior wall” technique [21], whereas others preformed the “through-and-through” technique [24]. Since the carpal bones are just underneath the artery and because puncture of the periosteum causes significant pain, the “through-and-through” technique is not recommended by some interventionalists [21]. After successful puncture, a 0.025-in. straight wire is inserted. When the vessel is tortuous and the 0.025-in. straight wire cannot be placed into the vessel, a 0.014-in. guidewire can be used to increase the success rate of cannulation [25]. Whether patients have been selected before the procedure, also has a certain impact on the success of the puncture. For cardiac catheterization with the dTRA, ultrasound facilitates selection of a suitable sheath by measuring the diameter of the artery in the AS [31]. In addition, the utilization of ultrasound allows the identification of anatomical landmarks and enables accurate vessel access [47]. Therefore, the success rate may be elevated under ultrasound guidance. In our previous experience, we tried the dTRA for cardiac catheterization using the “through-and-through” technique in 34 consecutive patients with palpable pulsation in the AS. After puncture, a 0.025-in. straight wire was used in our procedure. The success rate of puncture was 91.18% (31/34), and the rate of successful cannulation was 85.29% (29/34).

Although the diameter of the DRA was relatively small compared to conventional radial artery, most patients can be planted 6Fr sheath via DRA to complete the cardiac intervention. Gasparini GL et al. [33] shared their success experiences with left dTRA for coronary chronic total occlusion intervention in 41 patients using 7 Fr Glidesheath Slender. The novel technique was not only successfully applied in patients with stable angina pectoris, but also in patients with ST- elevated acute myocardial infarction [48] and unprotected true left main bifurcation lesion dealing with two-stent technique [49]. Once the puncture is successful, most of the cardiac catheterization can be completed without cross-over to other approaches. With the development of material technology, the widely use of hydrophilic sheath and sheathless catheters, the diameter of vessel is not the decisive factor for coronary intervention via the dTRA.

## Complications

As expected, the dTRA for cardiac intervention has superior safety and satisfaction [28]. As a novel approach for cardiac catheterization, access related complications should also be considered by operators, such as RAO, radial spasm, bleeding and haematoma, and injury of the superficial branch of the radial nerve [27, 29, 37, 39].

### RAO

Stenosis or occlusion after catheterization via the TRA will affect the future utilization of the radial artery, which becomes one of the important reasons for interventionalists to find alternative approaches. Post-catheterization stenosis and occlusion of the radial artery are common and related to several factors, including female sex, age, manual compression, and radial artery diameter [50]. Wakeyama et al. [51] found radial artery hypertrophy after transradial intervention by IVUS. After 6 months, the intima-media volume in patients who underwent transradial artery intervention was increased significantly, whereas the lumen volume and vessel volume decreased significantly. Recently, post-catheterization impairment of the radial artery, including intimal dissection, medial calcification, intimal injury, medial hypertrophy and adventitial neovascularization, was observed via optical coherence tomography at different time periods [37]. The incidence of distal radial artery occlusion (dRAO) was relatively low according to recent literature, ranging from 0.0–5.2% [29, 39]. In a large retrospective study, the incidence of dRAO was reported to be only 0.61% (10/1661) [27]. In another large retrospective study conducted in the Russian Federation, the total rate of dRAO was 2.2% (22/1009) in the AS approach [37]. The distribution of occlusion sites was 0.1% in the forearm radial artery, 1.8% in the AS, and 0.3% both in the forearm and the AS. The rate of occlusion in forearm via dTRA was decreased by 90% compared with that via the TRA approach (0.4% vs. 4.2%). However, the rate of occlusion was reported to be approximately 5% in a randomized study with a sample size of 100 [40]. Endothelial functions detected by ultrasound via a flow-mediated vasodilatation test compared with TRA were found to be significantly preserved or less influenced in the left DRA [52]. The low occlusion rate in the DRA may be explained by the following reasons. The artery in the AS is small and superficial, with a bony platform beneath it. Haemostasis does not require too much pressure for the compression device or bandage. Additionally, the compression time is greatly shortened with the DRA compared with the TRA [40, 42]. Why was the prevalence of forearm radial artery occlusion lower in the dTRA than in the TRA? One of the reasons may be that the haemostatic device is limited to compressing the vessel in the AS region. Second, the anatomical features of the distal radial artery are thought to contribute to it. During haemostasis, antegrade flow through the superficial palmar arch can be maintained even after occlusion in the DRA by reducing the risk of retrograde thrombus formation in the forearm radial artery [21]. Intermittent compression of the ipsilateral ulnar artery after pulling the DRA sheath to promote antegrade flow through the radial artery, can minimize the risk of radial artery occlusion. It seems a good idea [29]. What deserves attention is that the rate of DRA occlusion may increase with time after the procedure. Researchers have found that the rate of occlusion might slightly increase 1 month after the procedure compared to 24 h after the procedure [25, 33], which may be associated with the vessel remodeling.

Because most of the studies had small sample sizes and retrospective designs, conclusions should be made cautiously. Further randomized controlled and long-term follow-up studies with large sample sizes should be conducted to clarify the reasons for occlusion and how to reduce it.

### Bleeding, haematoma and pseudoaneurysm

Due to the structure of the AS with a bony basement surrounded by tendons, the incidence of serious bleeding, pseudoaneurysm and haematoma is not common. Whether a haemostastic device or bandage, haemostasis in the AS region is achieved more easily and quickly than that in the forearm [29, 40, 42], which can reduce hospital stay time and nursing work load in daily nursing practices [53]. In patients undergoing CAG, haemostasis can be achieved by finger compression of the puncture site for 15 min. Even for PCI, finger pressure can achieve haemostasis in patients with an ACT < 250 s at the end of the procedure [29].

Although the minor hematoma occurs sometimes, the prevalence of major haematoma is actually very low. The prevalence of haematoma more than 10 cm was reported only 0.2% in a large retrospective study [37]. Haematoma was likely precipitated by improper position of compression, using of dual antiplatelet drugs and heparin, old age, fragile skin, and multiple puncture attempts [25]. A 63 years old female undergoing PCI, occurred a serious haematoma in her hand after success haemostasis, which presented swelling, hand pain and restricted motion of her fingers. After using intermittent blood pressure cuff inflations, haematoma was gradually stable [54].

Pseudoaneurysm was extremely rare. In 2019, Prejean SP et al. [55] reported a case of pseudoaneurysm in the left DRA occurring 20 h after the sheath being removed out, which was cured by another compression.

### Numbness

Theoretically, the space of the AS is narrow and the superficial branch of the radial nerve nears to radial artery in the AS. Repeated puncture in the AS and long duration of compression time can damage the superficial branch of the radial nerve, leading to the numbness of digitals. However, the clinical reports about the numbness were rare [25]. In a real-world prospective observational study, Lee et al. [25] reported 2 out of 141 cases (1.4%) suffering from the numbness. Numbness in the fingers in 2 cases (1.0%) was also observed in a Japanese multicenter study [30]. Ultrasound-guided puncture can elevate the success rate and reduce the puncture attempts, which might facilitate to reduce the incidence of numbness.

### Pain

**Fig. 2:**
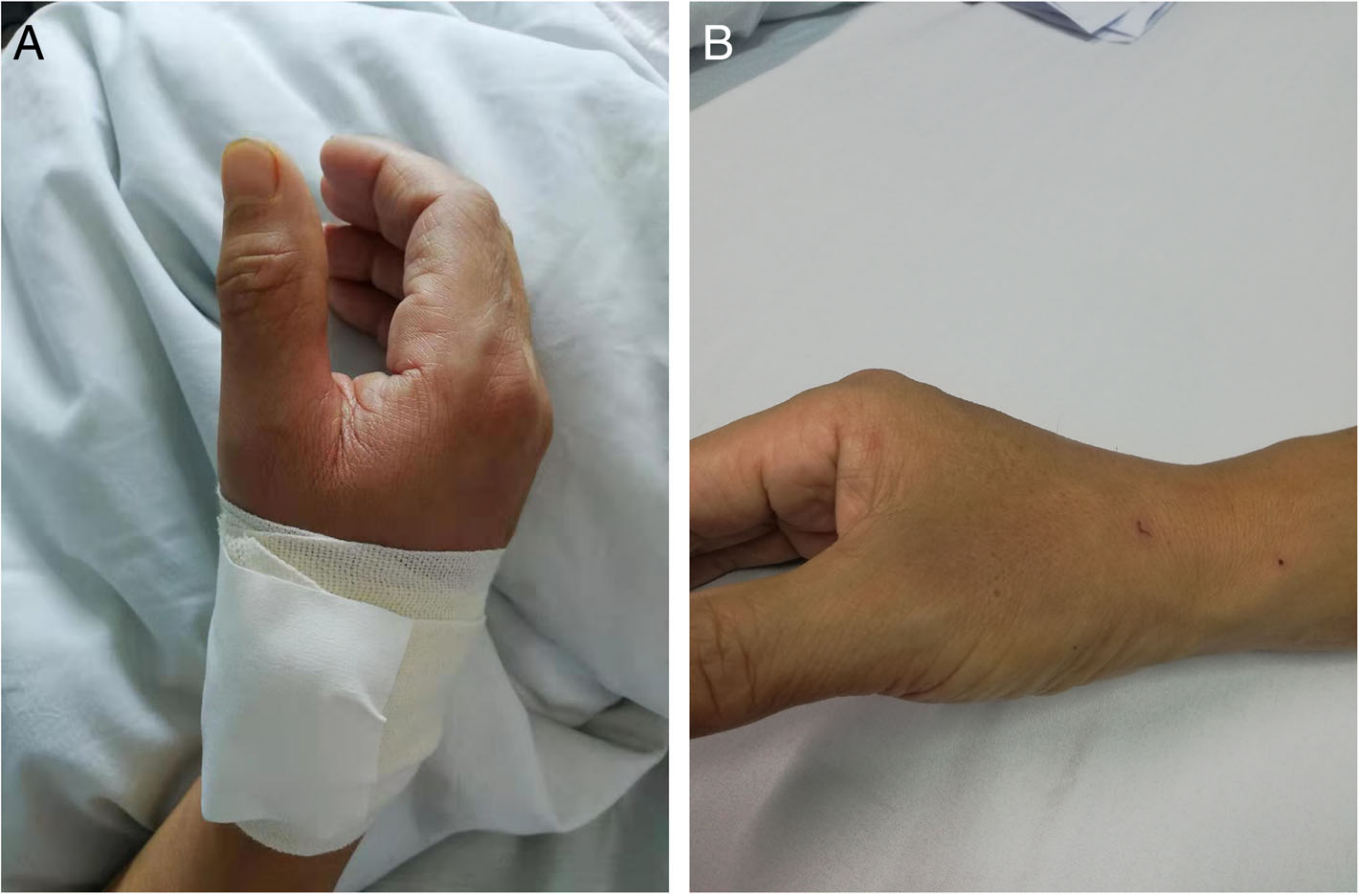
Hemastasis of dTRA (**a**. Hemastasis by bandage; **b**. Release of the bandage). (original image)

When using the TRA approach, the hand of the patient puts in supine or semi-supine position during the operation, which leads to patient discomfort. Especially when taking the left hand and the patient is obese, the procedure is depressing inevitably. Pain can reduce the patient’s satisfaction in the procedure. In practice, we found the blocked blood flow could lead to hand great swelling and pain, especially in patients compressed the TRA puncture site using bandage. However, the hand needs only to put in a natural position when through dTRA during the operation. Due to the special anatomic structure, compression after withdrawing the sheath does not need to block the blood flow completely, and the hemostatic time is relatively short (Fig. 2). All of the changes can also increase the patient’s satisfaction. In a case-control study, researchers investigated the satisfaction in patients. They found that the satisfaction in AS group was 89%, which was slightly higher than that in TRA group (87%) [42]. El Tahaan M, et al. [43] reported the satisfaction in AS group was significant higher than that in TRA group (90% vs. 72%). But the difference in left and right hands was not significantly. In some studies, the patient’s satisfaction was semi-quantized evaluated by using visual analog scale (VAS) [22, 56]. The VAS score of pain was lower in dTRA group in comparison to TRA group [56]. Al-Azizi KM et al. [28] reported that the novel approach could increase the satisfaction not only in patients, but also in operators and nurses.

## Application of ultrasound in the dTRA

### Measurement of the diameter of the DRA

Ultrasound can offer the accurate parameters of the radial artery in the AS to help interventionalists select the approach of the procedure. Among the published literature, the diameter of the DRA varies greatly [30, 57] (Table 2). In a Japanese retrospective study, the diameter of the DRA in the AS defined as the distance from media to media was 2.02 ± 0.44 mm, which was smaller than that of the conventional radial artery (2.57 ± 0.58 mm) [57]. The largest artery diameter in the AS reported by Norimatsu K was 2.6 ± 0.5 mm in the total population, 2.6 ± 0.5 mm in males, and 2.5 ± 0.5 mm in females, respectively [31]. However, the definition of vessel diameter was the distance from the lower edge of the adventitia of the vessel proximal wall to the upper edge of the adventitia of the vessel distal wall. In a prospective observational registry trial in Korea, the diameter of the DRA in the AS was 2.41 ± 0.50 mm in the left hand and 2.36 ± 0.49 mm in the right hand [25]. Although most studies concluded that the diameter of the DRA was smaller than that of the TRA, we cannot take it for granted that diameter of the DRA must be less than that of the TRA. Some studies found that there was no significant difference in diameter between the DRA and TRA [23, 29]. In addition, the variation was observed in 6.7% of subjects with a larger DRA than TRA [57]. Our initial experience also found this variation. Why is there a great difference in the diameter of the DRA among studies? One of the reasons is due to the difference in ethnicity, and the other is the difference in measurement methods, including the definition of the distance of diameter and the selected site of measurement. Measurement by the same skilled ultrasound expert and using the unified measurement methods may be beneficial to reduce the difference.

The gender difference in the diameter of DRA is significant. Such as, women in Korea [58] had a significant smaller diameter of DRA than men (2.40 mm vs. 2.65 mm, *P* = 0.016), which was also confirmed in Georgian population [29].

An interesting phenomenon was observed by vascular ultrasonography in the diameter during post-procedure follow-up [30]. Mizuguchi Y et al. [30] reported that the inner diameter of the DRA before procedure was 2.3 ± 0.4 mm, which was significantly enlarged to 2.7 ± 0.5 mm in 1 day after procedure and followed by reducing to near baseline levels (2.5 ± 0.5 mm) after 1 month. Recently, we also found the diameter in the AS after operation was larger than that before operation in some patients. This phenomenon even exited in patients with the ratio of DRA/ sheath outside diameter more than 1.0.

Whether the diameter of vessel is related to certain factors and can be predicted by simple methods is inconsistent. Although the diameter of the DRA was strongly associated with conventional radial artery (r = 0.68) [57], it varied greatly and could not predict by patient characteristics, such as age, gender, height, weight, body mass index, etc. However, Norimatsu K et al. [31] found that the diameter of the DRA was positively correlated with both body weight (*r* = 0.248, *p* = 0.003) and body mass index (*r* = 0.228, *p* = 0.007), although it was not correlated with age or height. Up to now, we cannot hastily conclude that the diameter of the DRA can be predicted.

### Advantages of using ultrasound in the perioperative period

Under the guide of ultrasound, the success rate of first puncture in traditional radial artery site can be increased compared to digital palpation technique [59]. Similarly, ultrasound guided puncture not only facilitated to increase the successful rate of puncture, but also reduce the complications of puncture. Although the application of thin-wall sheaths could reduce the outside diameter of the sheath reserving the large inner size, the ratio of sheath outside diameter/ DRA was **≥**1.0 in part patients inevitably, which could increase the damage of endothelium, RAO and pain during the procedure. Measurement of the diameter of the DRA before cardiac catheterization is important, which may be superior to pulsation only [31]. The narrow triangular region contains some important structures, such as the DRA, the superficial branches of the radial nerve and the cephalic vein. During the operation, ultrasound guidance can identify important anatomical landmarks and avoid injuring some important structures near the radial artery, which can reduce puncture-related complications [47]. After the procedure, ultrasound can detect occlusion of the DRA. According to previous studies, the rate of occlusion of the DRA is relatively small. However, the long-term ultrasound evaluation data remain inadequate.

## Application in cerebral angiography and intervention

In addition to CAG and PCI, the dTRA is also applied for other interventional diagnoses and treatments [60–62]. For example, dTRA was certified a feasible and safe technique for abdominal interventional radiology embolization procedures [62]. In recent years, the advantages of the dTRA for vascular intervention have also attracted the attention of neurointerventionalists (Table 3) [63–67]. In 2018, McCarthy D et al. [63] first reported the successful experiences in two patients. One patient only underwent a cervical angiogram with a 5Fr sheath, and the other underwent mechanical thrombectomy and balloon angioplasty of the basilar artery with 088″ infinity guide catheter. No access-related complications were observed. Al Saiegh F et al. [64] successfully implanted the Woven EndoBridge device in a middle-aged female suffering from an anterior communicating aneurysm via the dTRA. Two retrospective studies with 116 patients undergoing diagnostic cerebral angiography were reported in 2019 [65, 67]. The total success rate of cannulation via the dTRA in these studies was 90.52%. A non-randomized study including 58 cases via the dTRA and 151 via the TRA, was conducted to explore the feasibility and safety of the novel technique in carotid intervention using 6.5F JR5 sheathless guiding [66]. The procedural success rate was not significantly different in the dTRA group (100%) and TRA group (94%). In contrast to the TRA group, the fluoroscopy and procedure times were significantly higher in the dTRA group. However, there was no difference in the contrast consumption and cumulative X-ray dose between the two groups. Only one case of AV fistula requiring surgical reconstruction in the dTRA group and two cases of asymptomatic RAO in the TRA group were observed.

**Table 3:** Characteristics of researches on the feasibility of dTRA in cerebral angiography and intervention

Year	First author	Country	Study design	Age (Mean, y)	Sample size (n)	Male (%)	A/I	Glidesheath	Success rate (%)
2018	McCarthy DJ [63]	American	Case report	NA	2	NA	A/I	5F Glidesheath Slender/0.88″ sheathless guide catheter	100.0
2019	Al Saiegh F [64]	American	Case report	53	1	0.0	I	5French short sheath	100.0
2019	Brunet MC [65]	American	Retrospective	53.8	85	21.2	A	5F Glidesheath Slender (Terumo, Japan)	91.8
2019	Nardai S [66]	Hungary	Retrospective	NA	58	NA	A/I	4-5F transradial sheaths (Terumo Co., Japan)/6.5F JR5 sheathless guiding (Asahi, Japan)	100.0
2019	Patel P [67]	American	Retrospective	54.5	31	50.0	A	5F Prelude Ideal (Merit Medical, South Jordan, Utah) or 5F Glidesheath Slender (Terumo, Somerset, New Jersey)	87.10

*Abbreviations*: *A* angiography, *I* intervention, *NA* not available

The novel approach used in cerebral intervention is more challenging for neurointerventionalists, due to the torturous and smaller anatomy of cerebral vessels. The feasibility and safety of the dTRA in cerebral angiography and intervention needs to be verified by large-sample and randomized studies in the future.

## Conclusions

From the viewpoint of radial artery preservation and patients’ comfort, the DRA is undoubtedly the best alternative choice for conventional radial artery approach. However, in clinical practice, there are still some problems which need to be solved urgently. If can all patients with palpable artery pulse in AS undergo cardiac catheterization by using DRA? If is dTRA suitable for application in patients with acute myocardial infarction? In addition, the length of the catheter may not be sufficient in tall patients or in patients with tortuous artery, which leads to change the approach site. At last, special haemostatic devices based on local structural features should be designed.

Nowadays, it is undeniable that the TRA remains the preferred selection for cardiac catheterization in most patients. Just as DR Corcos T [68] expected: radial is better, smaller is better, distal radial is even better! With the development of material technique of sheath and guiding catheters, the dTRA becoming the default for cardiac catheterization is no longer a dream.

## Data Availability

The datasets used and/or analysed during the current study available from the corresponding author on reasonable request.

## Abbreviations

TRA: Transradial access
RAO: Radial artery occlusion
dTRA: Distal transradial approach
CAG: Coronary angiograph
PCI: Percutanous coronary intervention
DRA: Distal radial artery
AS: Anatomic snuffbox
